# Community engagement around scrub typhus in northern Thailand: a pilot project

**DOI:** 10.1093/trstmh/trae028

**Published:** 2024-05-06

**Authors:** Carlo Perrone, Nipaphan Kanthawang, Phaik Yeong Cheah, Daranee Intralawan, Sue J Lee, Supalert Nedsuwan, Benjarat Fuwongsitt, Tri Wangrangsimakul, Rachel C Greer

**Affiliations:** Mahidol-Oxford Tropical Medicine Research Unit, Faculty of Tropical Medicine, Mahidol University, Bangkok 10400, Thailand; Centre for Tropical Medicine and Global Health, Nuffield Department of Medicine, University of Oxford, Oxford OX3 7LG, UK; Mahidol-Oxford Tropical Medicine Research Unit, Faculty of Tropical Medicine, Mahidol University, Bangkok 10400, Thailand; Mahidol-Oxford Tropical Medicine Research Unit, Faculty of Tropical Medicine, Mahidol University, Bangkok 10400, Thailand; Centre for Tropical Medicine and Global Health, Nuffield Department of Medicine, University of Oxford, Oxford OX3 7LG, UK; Social and Preventive Medicine Department, Chiang Rai Prachanukroh Hospital, Chiang Rai 57000, Thailand; Mahidol-Oxford Tropical Medicine Research Unit, Faculty of Tropical Medicine, Mahidol University, Bangkok 10400, Thailand; Centre for Tropical Medicine and Global Health, Nuffield Department of Medicine, University of Oxford, Oxford OX3 7LG, UK; Social and Preventive Medicine Department, Chiang Rai Prachanukroh Hospital, Chiang Rai 57000, Thailand; Social and Preventive Medicine Department, Chiang Rai Prachanukroh Hospital, Chiang Rai 57000, Thailand; Mahidol-Oxford Tropical Medicine Research Unit, Faculty of Tropical Medicine, Mahidol University, Bangkok 10400, Thailand; Centre for Tropical Medicine and Global Health, Nuffield Department of Medicine, University of Oxford, Oxford OX3 7LG, UK; Mahidol-Oxford Tropical Medicine Research Unit, Faculty of Tropical Medicine, Mahidol University, Bangkok 10400, Thailand; Centre for Tropical Medicine and Global Health, Nuffield Department of Medicine, University of Oxford, Oxford OX3 7LG, UK; Nuffield Department of Primary Care Health Sciences, University of Oxford, Oxford OX2 6GG, UK

**Keywords:** community engagement, health literacy, prevention and control, scrub typhus

## Abstract

**Background:**

Scrub typhus is highly endemic in northern Thailand yet awareness and knowledge are low. We developed a community engagement project to improve awareness in communities at risk of contracting scrub typhus.

**Methods:**

We conducted a series of engagement sessions with healthcare workers and community health volunteers so they would, in turn, engage with their communities. We evaluated our activities by assessing the increase in scrub typhus knowledge, using a series of Likert-scale items and open-ended questions. Three to 6 months after the sessions, participants were followed up to collect their experiences training community members.

**Results:**

Of 134 participants who took part in eight sessions, 87.3% were community health volunteers. Disease knowledge increased substantially after the sessions and was well maintained for up to 5 mo. Satisfaction was high and, through participant feedback, engagement materials were improved to be more useful to the communities. People with higher education had higher scores and retention.

**Conclusions:**

Community engagement was shown to be an effective tool to develop and carry out health-promoting activities in a culturally and context-appropriate manner.

## Introduction

Scrub typhus is a potentially fatal cause of acute febrile illness, characterised by symptoms such as headache, malaise, rash and abdominal pain. Eschars, characteristically painless, necrotic skin lesions, are present in a variable proportion of cases but are highly specific for clinical diagnosis. Scrub typhus is common in south and southeast Asia.^1^ However, it is hard to distinguish from other diseases due to its mostly non-specific symptoms and lack of reliable point-of-care diagnostic tests. These challenges can result in delayed diagnosis and treatment.^2^

Humans develop scrub typhus when bitten by infected trombiculid mite larvae, which transmit *Orientia tsutsugamushi* (or *Candidatus* O.chiloensis and *Candidatus* O.chuto), the causative agent. These larval mites, or chiggers, can be found near rivers, forests, tall weeds and grasses, and typically feed on rodents.

Changing work clothes and showering at the end of the day, avoiding exposure of naked skin and prolonged contact with vegetation are likely to reduce the risk of infection. The preventative effect of such behaviours is supported by case-control studies and there are increasing calls for educational programmes so that individuals can reduce their risk of contracting scrub typhus.^3–7^

In Thailand, almost one-half of the reported cases are from the northern region; Chiang Rai Province reports >700 cases a year.^8^ Despite this, awareness among patients and community health volunteers (CHVs) is low and the majority of patients admitted to our linked hospital with scrub typhus have never heard of the disease.^9^

Nearly one-half of the population in Chiang Rai Province work in agriculture,^10^ commonly in hilly and forested areas that are often inhabited and farmed by a number of ethnic minorities known as ‘hilltribes’, who are disproportionately affected by the disease.^9^ They generally have a lower socioeconomic status and encounter several barriers to healthcare access, including language barriers and distance from healthcare.^11,12^ Each group has its own traditions and language and many of their members do not speak Thai fluently, which can make standard health promotion and education challenging. There is a paucity of scrub typhus educational materials globally and, to the best of our knowledge, there are none available in hilltribe languages.

Community engagement can be used to raise awareness and share research benefits, including new knowledge with communities. It can therefore improve health and change health behaviours, especially when it is collaborative and involves two-way learning.^13,14^ CHVs, members of the community who received some healthcare training, have been shown to be effective in sharing research knowledge and providing health education.^14,15^ They can be especially valuable in low- and lower middle-income settings, where resources for health education are limited and where context- and culturally appropriate strategies are especially needed for disadvantaged groups.^14,16,17^

Thailand is divided into provinces (each having a provincial hospital), provinces are divided into districts (each having a district hospital) and districts are divided into subdistricts (each hosting a primary care unit [PCU], also known as a ‘subdistrict health promoting hospital’). PCUs therefore represent the first point of access to healthcare for communities; they are staffed by nurses and public health officers, herein referred to as healthcare workers (HCWs), and provide primary care and preventative services. Each PCU manages a network of 20 to 30 CHVs, who receive a modest financial allowance and provide a link between communities and PCUs. Their main duties are health promotion and maintaining family health records for the households within their catchment area.

To raise awareness of scrub typhus, we engaged with CHVs and HCWs in PCUs with a view to (i) understand their current knowledge of scrub typhus; (ii) provide and adapt context-specific training through engagement; and (iii) evaluate the effectiveness of this approach through changes in scrub typhus knowledge, participant satisfaction and training experiences.

## Materials and Methods

### Project setting

The project was carried out in PCUs in Chiang Rai Province, northern Thailand. Approximately 20% of the population of Chiang Rai belong to a hilltribe ethnic minority group such as Akha, Lahu and Karen.^18^

We targeted five PCUs with the highest number of reported scrub typhus cases in the central Mueang District of Chiang Rai Province. Each PCU director was asked to invite up to 25 CHVs and as many HCWs working in the PCU as possible by convenience sampling. All participants were aged ≥18 y. There were no formal exclusion criteria. However, CHVs were preferentially selected if they could understand and communicate in Thai.

### Engagement approach

#### Training materials

Training materials consisted of a 15-page flipchart with illustrations and a 6.5-min video.^19,20^ The first versions of the flipcharts and video were designed by researchers from the project team and local primary care doctors. They feature artwork and photography commissioned from local artists, and depict key messages on scrub typhus epidemiology, clinical features, management and preventative behaviours. We took care to faithfully represent the local context in the artwork. All materials were presented in central Thai; in addition, the video was translated and dubbed into Akha and Lahu languages. As part of the engagement process, the materials were adapted and new materials were developed following feedback from participants.

#### Training and engagement session, and follow-up

Participants attended a single half-day session at a local PCU that was conducted in central or northern Thai dialects. HCWs and CHVs attended separately. An interactive presentation was followed by a review and demonstration of the materials. Participants were then asked to role play training each other using the flipcharts in small groups of four to six. Each session concluded with participants being asked to teach villagers or patients under their care what they had learnt with a particular focus on disease recognition and prevention and were given access to the materials for use at their own discretion.

Participants were followed up in person, or by telephone if they were unable to attend in person, 3–6 mo later. Only one participant required follow-up by telephone; as they had missed part of the training, their results were not included in the final analysis.

#### Evaluation of the engagement approach

Activities were evaluated through:

1- A self-administered questionnaire to assess scrub typhus knowledge, consisting of 13 multiple-choice questions with a single correct answer relating to disease epidemiology, clinical features and management. It was created by local doctors and researchers, piloted with a group of CHVs (from PCUs in Chiang Rai that were not part of the engagement programme) and revised for clarity. It was filled out before and after the training session and at the 3–6 mo follow-up. The increase in knowledge and its maintenance at 3–6 mo were used to evaluate the effectiveness of the engagement approach.
2- A self-administered evaluation form to assess satisfaction with the training, completed immediately after the training. This contained Likert-scale questions on aspects of the training (e.g. the venue, speaker and the materials) and open questions for general recommendations.
3- A series of self-administered closed and open-ended questions, collecting participants’ training experiences, at the 3–6 mo follow-up.
4- Informal discussions with participants, who were encouraged to share their knowledge and experience of scrub typhus and to discuss ways of passing on the information to villagers and to identify potential barriers towards the preventative measures.
5- Team debriefs, after each session, to discuss what went well, what could be improved and to ensure that two-way engagement took place.

Fieldnotes were taken to capture practical details, informal feedback and reflections.

If participants were unable to read or speak Thai fluently, an assistant translated and/or helped them complete the evaluation processes.

#### Analysis

Participants were included in the analysis if they attended the full training session and the follow-up appointment. Knowledge scores were calculated from the responses to the scrub typhus knowledge questions, each scoring one point if correct, for a total of 13. Test scores were summarised using medians and 25th–75th percentile (p25, p75). Pre-session scores were compared between groups using the Kruskal–Wallis test. Associations with variables that were significant at p<0.05 were then quantified in multivariable regression models. When variables were collinear, only one was selected for inclusion according to model fit (Akaike's information criterion). Models using the difference in scores were adjusted for pre-training test scores (i.e. difference from pre- to post-test) or both pre- and post-training test scores (i.e. difference from post-test to follow-up). Data were analysed using STATA version 17 (College Station, TX, USA).

## Results

### Participants’ demographics and scrub typhus knowledge before training

In total, 141 participants took part in eight sessions (five for CHVs and three for HCWs) from June to September 2020. Of these, four participants missed part of the training session and three were lost to follow-up, resulting in 134 participants available for analysis. The majority of participants were CHVs (117/134, 87.3%), who were predominantly farmers (Table 1). Their median age was 41 y (p25, p75: 34, 49) and one-half of participants were female. Nearly 70% of participants were non-Thai (69.4%), with the Akha and Lahu being the most represented ethnic groups, together accounting for >50% of participants.

**Table 1.** Demographic details of the participants and their pre-session, post-session and follow-up scrub typhus knowledge scores.

Demographic details	Number of participants, n/N (%)	Median pre-session knowledge score (p25, p75)	Median post-session knowledge score (p25, p75)	Median 3–6 mo follow-up knowledge score (p25, p75)
Total	N=134	7 (4, 9)	13 (12, 13)	12 (11, 13)
Healthcare worker role, n (%)		*p<0.001*		
Nurse	6/134 (4.5%)	12 (10, 13)	13 (13, 13)	13 (13, 13)
Other HCW	11/134 (8.2%)	9 (8, 11)	13 (12, 13)	13 (12, 13)
CHV	117/134 (87.3%)	7 (4, 9)	13 (12, 13)	12 (11, 12)
Occupation, n (%)		*p<0.001*		
Farmer	71/134 (53.0%)	7 (3, 9)	13 (12, 13)	12 (11, 12)
Daily labourer	14/134 (10.4%)	6 (6, 9)	13 (11, 13)	12 (11, 12)
Merchant	11/134 (8.2%)	7 (6, 9)	13 (11, 13)	11 (11, 12)
Healthcare worker	17/134 (12.7%)	10 (9, 12)	13 (13, 13)	13 (12, 13)
Other	21/134 (15.7%)	7 (5, 8)	12 (12, 13)	12 (11, 12)
Gender, n (%)		*p=0.27*		
Female	67/134 (50.0%)	8 (5, 10)	13 (12, 13)	12 (11, 13)
Male	67/134 (50.0%)	7 (4, 9)	13 (12, 13)	11 (11, 12)
Median age (IQR), y	41.0 (34.0, 49.0)	7 (4, 9)	13 (12, 13)	12 (11, 13)
Age group, n (%)		*p=0.45*		
<35 y	35/134 (26.1%)	7 (5, 10)	13 (12, 13)	12 (11, 13)
35 to 39 y	23/134 (17.2%)	6 (3, 9)	13 (12, 13)	12 (11, 12)
40 to 49 y	44/134 (32.8%)	8 (5.5, 9)	13 (12, 13)	12 (11, 13)
≥50 y	32/134 (23.9%)	7 (3, 9)	13 (11, 13)	11 (10, 12.5)
Educational level, n (%)		*p<0.001*		
Primary school or less	9/133 (6.8%)	2 (2, 5)	13 (12, 13)	11 (11, 11)
Primary school certificate	33/133 (24.8%)	7 (4, 9)	12 (11, 13)	12 (10, 13)
High school certificate	69/133 (51.9%)	7 (4, 9)	13 (12, 13)	12 (11, 12)
Higher education	22/133 (16.5%)	10 (9, 12)	13 (13, 13)	13 (12, 13)
Ethnicity, n (%)		*p= 0.002*		
Thai	41/134 (30.6%)	8 (6, 10)	13 (11, 13)	12 (11, 13)
Akha	37/134 (27.6%)	5 (2, 7)	13 (12, 13)	11 (11, 12)
Lahu	32/134 (23.9%)	7 (5, 9)	12 (12, 13)	12 (11, 13)
Karen	16/134 (11.9%)	9 (8, 10)	13 (12.5, 13)	12 (11, 13)
Other	8/134 (6.0%)	9 (3.5, 11)	13 (12, 13)	11.5 (11, 12)
Belief that scrub typhus is a problem in this area, n (%)		*p≤0.001*		
Agree	52/131 (39.7%)	9 (7, 11)	13 (12, 13)	12 (11, 13)
Neutral/don't know	62/131 (47.3%)	5.5 (2, 8)	13 (12, 13)	12 (11, 12)
Disagree	17/131 (13.0%)	7 (6, 8)	12 (12, 13)	11 (10, 12)

p25, p75: 25th and 75th percentile; *p-values* compare pre-session knowledge scores between categories using the Kruskal-Wallis test.

**Figure 1.**
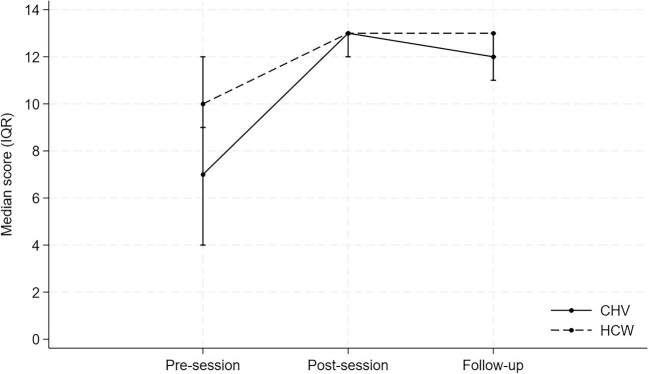
Median pre-session, post-session and follow-up scrub typhus knowledge scores, by healthcare workers (HCW) and community health volunteers (CHV).

Before training, scrub typhus knowledge was low, with a median score of 7 (4–9) out of 13. Median scores increased to 13 (12–13) after the training session and were maintained at 12 (11–13) at the 3–6 mo follow-up (Figure 1 and Table 1). Of note, nine CHVs scored zero pre-session but their scrub typhus knowledge increased to 13 post-session (12–13). The individual pre-session knowledge questions and responses are shown in Supplementary Table S1.

Multivariable analysis indicated that existing knowledge of scrub typhus was likely to be higher in participants who had attained higher levels of education; those with higher education scored, on average, 5.3 points higher (95% CI 2.22 to 8.29) than those who had not completed primary school on the knowledge questionnaire before the training session (Table 2). Participants who did not believe that scrub typhus was a problem in the area scored, on average, 2.2 points lower (95% CI −3.39 to −1.06).

**Table 2.** Multivariable model of factors associated with pre-session scrub typhus knowledge score.

Variable	Coefficient (95% CI)	p
Education		
Less than primary school	Reference	
Primary school certificate	2.36 (0.15 to 4.56)	0.037
High school certificate	2.59 (0.50 to 4.68)	0.016
Higher education	5.26 (2.22 to 8.29)	0.001
Ethnicity		
Thai	Reference	
Akha	−1.60 (−3.46 to 0.26)	0.091
Lahu	−1.20 (−3.22 to 0.83)	0.245
Karen	−0.13 (−2.37 to 2.10)	0.906
Other	−0.46 (−3.10 to 2.18)	0.732
Belief that scrub typhus is a problem in this area		
Agree	Reference	
Disagree	−2.23 (−3.39 to −1.06)	<0.001
Don't know or neutral	−0.80 (−2.48 to 0.89)	0.350
Self-reported awareness of scrub typhus pre-session	1.14 (−0.12 to 2.40)	0.075
Self-reported experience of scrub typhus patients pre-session	0.33 (−1.15 to 1.80)	0.662
Healthcare worker (rather than CHV)	−0.23 (−2.80 to 2.34)	0.860
PCU workplace		
PCU 1	Reference	
PCU 2	−1.41 (−3.45 to 0.63)	0.174
PCU 3	2.12 (0.17 to 4.07)	0.033
PCU 4	−0.61 (−2.32 to 1.11)	0.485
PCU 5	0.67 (−1.24 to 2.57)	0.489

Abbreviation: PCU, primary care unit.

### Evaluation of the project activities

#### Effect of the training on knowledge scores

Test scores improved from pre- to post-session, with a median of 5 (3–8) more questions being answered correctly. On multivariable analysis, only the pre-session score was significantly associated with a change in test scores. Location of training (PCU) was borderline significant, with a smaller difference between pre- and post-session knowledge scores for one PCU (p=0.038) when compared with the reference PCU (Supplementary Table S2). Univariate analysis results are presented in Supplementary Table S3.

Participants’ knowledge was well maintained 3–6 mo after the sessions. The median score at follow-up was 12 (11–13) compared with 13 (12–13) immediately after the training.

When included in a multivariable model, participants’ education level and score on the post-test were the strongest predictors of score maintenance (Table 3).

**Table 3.** Multivariable analysis of the paired difference between post-session and follow-up scrub typhus knowledge scores.

Variable	Coefficient (95% CI)	p
Pre-session knowledge score	−0.02 (−0.10 to 0.06)	0.583
Post-session knowledge score	−0.59 (−0.84 to 0.34)	<0.001
Education		
Less than primary school	Reference	
Primary school certificate	1.12 (0.78 to 2.17)	0.035
High school certificate	1.21 (0.21 to 2.21)	0.018
Higher education	1.63 (0.14 to 3.11)	0.032
Healthcare worker (rather than CHV)	0.67 (−0.49 to 1.84)	0.253
PCU of training		
PCU 1	Reference	
PCU 2	0.67 (−0.28 to 1.63)	0.166
PCU 3	0.97 (0.04 to 1.91)	0.042
PCU 4	0.14 (−0.64 to 0.93)	0.720
PCU5	0.45 (−0.54 to 1.45)	0.368
Ethnicity		
Thai	Reference	
Akha	0.15 (−0.67 to 0.98)	0.714
Lahu	−0.37 (−0.95 to 0.88)	0.936
Karen	0.27 (−0.78 to 1.33)	0.606
Other	0.17 (−1.03 to 1.38)	0.782

Abbreviation: PCU, primary care unit.

#### Training session evaluation

Illustrative quotes from participants’ free text answers are used to demonstrate the findings. All 134 participants expressed high levels of satisfaction with the training (Table 4).

**Table 4.** Level of participant satisfaction with the training sessions (N=134).

	Very low, n (%)	Low, n (%)	Moderate, n (%)	High, n (%)	Highest, n (%)	Likert-score median (p25, p75)
Did you enjoy the training?	3 (2%)	4 (3%)	10 (7%)	43 (32%)	74 (5%)	5 (4, 5)
Satisfaction with:						
the venue	2 (1%)	1 (1%)	12 (9%)	38 (28%)	81 (60%)	5 (4, 5)
the speakers	2 (1%)	2 (1%)	5 (4%)	35 (26%)	90 (67%)	5 (4, 5)
the timing/length of the training	1 (1%)	3 (2%)	17 (13%)	49 (37%)	64 (48%)	4 (4, 5)
the content of the training	2 (1%)	1 (1%)	13 (10%)	44 (33%)	74 (55%)	5 (4, 5)
the format of the teaching	1 (1%)	3 (2%)	13 (10%)	42 (31%)	75 (56%)	5 (4, 5)
the chance to interact with others and ask questions	2 (1%)	2 (1%)	21 (16%)	51 (38%)	58 (43%)	4 (4, 5)
The teaching media is easy to understand	1 (1%)	2 (1%)	18 (13%)	47 (35%)	64 (47%)	4 (4, 5)

p25, p75: 25th and 75th percentile.

When asked what they liked, the most frequent response was (56/103, 54.4%): gaining new knowledge. Some went on to say that this knowledge would be useful and they would share it with others:

*Gained knowledge of how to protect oneself and be able to pass on knowledge to others* (PE133).

According to participants, the approachability of the speakers and the use of simple, clear language facilitated learning and knowledge transmission. The choice of training topic was also appreciated, yet several participants would have liked additional sessions on other topics or more time to practise using the materials.

#### Participants’ experiences of training others

Out of 134 participants, 125 (93.3%) reported training others after the session. Of these, 48/124 (38.7%) reported training >10 people (one missing response). The flipchart was used by 50/125 (40.0%), the video by 20/125 (16.0%). Of the 125 who reported training others, 116 responded to a question about using other methods to train, with 104 (89.7%) employing other methods in addition or in parallel to the materials provided (e.g. orally presenting at village meetings or small gatherings, using village loudspeakers).

Participants found the materials easy to understand (84% gave a Likert-scale score of 4 or 5; Table 4). The inclusion of pictures in the flipcharts and hilltribe languages in the video was felt to improve villagers’ understanding and helped those who struggled to read Thai.

*It's easy to use, look at the picture and explain accordingly, [I] can't really read Thai in the flipchart, so I explain through the pictures* (PE123).

*The pictures in the flipchart are something that made them understand more* (PE112).

However, there were challenges to using the materials. Initially, one large flipchart was given to each PCU for CHVs to borrow to teach their villagers, but this was not practical, especially for those who lived far away from the PCU. The flipchart was also bulky and difficult to transport on motorbikes. So, the flipcharts were not always used. A lack of internet meant that it was challenging to show the video to groups of villagers and CHVs were often reliant on their personal phones rather than larger screens:

*There was no projector [so I] opened [the video] on mobile phone, there were many people, the screen was small, couldn't see* (PE033).

*The teaching materials need internet, projector, etc., but the villagers don't have that equipment, the internet is not available* (PE134).

Participants suggested dubbing the video into other hilltribe languages, such as Karen and Yao; to provide a smaller version of it, easier to share on messaging apps; and to provide an audio track to be played over the village speakers.

The majority of the participants reported that they received positive responses to their teaching, despite most villagers never having heard of scrub typhus before:

*This [training] is very good because this disease is important, most people have never heard about scrub typhus before, the feedback was very good. They will prevent [protect] themselves and they also gained more knowledge* (PE132).

However, a few villagers were not interested because they did not believe that scrub typhus existed or they had never had it before. Others felt it could be treated by traditional medicine:

*Some older people said that…if someone had this disease, they didn't need to see a doctor, there was traditional folk wisdom treatment* (PE001).

A few participants felt that the villagers would receive teaching from doctors better than from CHVs and felt unable to teach or make recommendations. Others struggled to remember all the information when training communities:

*I don't know how to teach; I can't remember the content…The villagers were not interested in the CHV but they believe the doctor, they will ask the doctor themselves* (PE088).

### Engagement approach changes

As a consequence of the feedback, materials were changed and updated; a smaller flipchart and a pocket-sized leaflet were developed. In this way all CHVs had easily transportable personal materials. The audio tracks from the videos were also made available (in Thai, Akha and Lahu), to be played on village loudspeakers and reach communities easily.

## Discussion

CHVs had a relatively low baseline knowledge of scrub typhus. However, through our engagement approach, participants’ knowledge scores increased and were maintained at 3–6 mo.

Clear simple messages, using local languages, and repeated practice with the materials, helped achieve this. Constructive dialogue throughout the project between our team and the participants deepened our understanding and knowledge of the local context and health beliefs related to scrub typhus. This in turn allowed us to adapt our training approach and materials. Despite increasing recognition of the importance of scrub typhus and the need to increase communities’ awareness of it, we were unable to find reports of other educational programmes or community-engagement activities focusing on scrub typhus. Our findings show that such work is achievable and well received by those living in affected areas.

Participant satisfaction with the training was very high. Participants valued gaining knowledge about scrub typhus and being able to share this with others. More than 90% of participants went on to train others in their communities, sometimes using approaches we had not thought of, such as using village loudspeakers. Awareness of scrub typhus among villagers was low, the majority had not heard of it before, despite living in an endemic area. By comparison, 61%–80% of South Korean villagers had heard of scrub typhus.^7^

Our experience strongly supports work with CHVs. Their connection with the community, understanding of the context and ability to speak the local languages make them effective conduits between the community and healthcare institutions. This position is supported by a published review of CHVs’ roles that identified three main functions: health education, providing links to the community and clinical services.^21^ However, there is a need to provide ongoing political, financial, training and professional support, so that CHVs are empowered and valued by communities. Several CHVs in this project felt that doctors or researchers would be better suited to training villagers, which warrants further discussion and consideration, and may suggest the need for further training and support.

In line with community-based participatory research and culture-centred approach principles, we found that having a context- and culture-sensitive strategy helped with engaging participants.^22^ In developing our activities, we paid particular attention to culture (by employing local artists who depicted scenes of everyday life), language (northern Thai and hilltribe languages), trying to overcome obstacles (such as distance, by providing training at local PCUs; and transportation, by creating easily portable materials) and demographic factors (such as education and literacy levels, trying to use simple and clear terms and providing unwritten information). These elements were appreciated by participants and contributed to establishing mutual respect.

### Limitations

This was a pilot project, of limited scope and duration, where single engagement sessions were conducted with participants. Instituting a longer-term community engagement project with regular meetings would build stronger relationships, and deepen understanding of the health needs and difficulties that communities face. This would allow for repeated and more effective engagement activities on several topics with community-driven approaches.

To optimise resources and increase sustainability, we decided to focus on HCWs and CHVs, so that they would in turn disseminate knowledge to communities. Through a train the trainer approach, many people can be reached in an efficient and cost-effective manner.^23,24^ However, because we measured the effectiveness of our strategy on CHVs and HCWs (tier 1 trainees), not the effectiveness of their trainings on community members (tier 2), we assessed an intermediate rather than a final outcome (e.g. behavioural change in communities). A change in knowledge does not guarantee a change in behaviour and at times can have unexpected consequences.^25,26^ In addition, by using a self-administered questionnaire to assess the number of community members trained by participants, recollection or reporting bias may have occurred. External evaluation of the project would strengthen the findings.

#### Adaptations and implications for future work

Further engagement work is needed for scrub typhus and other neglected tropical diseases to raise awareness among affected communities and understanding among healthcare workers. We hope that our example will inspire future community engagement projects aiming to raise disease knowledge and equip communities. Future engagement projects should work directly with the target communities. Increasing accessibility for minority ethnic groups and those with limited literacy should be prioritised. Co-producing training materials with the anticipated end users could enhance the material and training package. The impact on health behaviour and outcomes should be evaluated. This may involve temporal analysis of scrub typhus incidence to the village level from disease surveillance data.^8^ Further work should be conducted to explore villagers’, and in particular, older people’s health beliefs towards scrub typhus and the role of traditional medicine. Experience with HIV supports the engagement of traditional healers, who do not necessarily consider themselves as alternatives but rather as complements to other forms of healthcare.^27,28^

### Conclusions

Scrub typhus is an important, yet an under-recognised disease. Our approach effectively engaged CHVs and HCWs in high-risk communities and increased scrub typhus knowledge and awareness, which we expect will improve disease management and prevention.

Two-way dialogue and clear communication strategies contributed to the success of our activities and helped us to understand the reality of what is happening on the ground and the challenges that CHVs and HCWs face. Further community engagement work is needed to raise awareness of scrub typhus and to evaluate its impact on villagers and their health behaviours.

## Supplementary Material

trae028_Supplemental_File

## Data Availability

The dataset will be made available from the MORU Data Access Committee upon reasonable request. A data access agreement will be put in place prior to data transfer. Instructions and the data application form are available at https://www.tropmedres.ac/units/moru-bangkok/bioethics-engagement/data-sharing. For the purpose of Open Access, the author has applied a CC BY public copyright licence to any Author Accepted Manuscript version arising from this submission.

## References

[1] Thumnu P, Uttayamakul S, Sangsajja C. Acute undifferentiated Febrile illness: a review of the studies in tropical countries reported during 2004 to 2015. J Med Tech Assoc Thailand. 2017;45:5898–5908.

[2] Paris DH, Dumler JS. State of the art of diagnosis of rickettsial diseases: the use of blood specimens for diagnosis of scrub typhus, spotted fever group rickettsiosis, and murine typhus. Curr Opin Infect Dis. 2016;29(5):433–9. 27429138 10.1097/QCO.0000000000000298 PMC5029442

[3] Kweon SS, Choi JS, Lim HS et al. A community-based case-control study of behavioral factors associated with scrub typhus during the autumn epidemic season in South Korea. Am J Trop Med Hyg. 2009;80(3):442–6. 19270296

[4] Lyu Y, Bie C, Dou X et al. Epidemiological characteristics of scrub typhus in Beijing during 2009-2019. Chinese Journal of Zoonoses. 2021;37(3):236–40.

[5] Rose W, Kang G, Verghese VP et al. Risk factors for acquisition of scrub typhus in children admitted to a tertiary centre and its surrounding districts in South India: a case control study. BMC Infect Dis. 2019;19(1):665. 31349809 10.1186/s12879-019-4299-2 PMC6660696

[6] Tran HTD, Hattendorf J, Do HM et al. Ecological and behavioural risk factors of scrub typhus in central Vietnam: a case-control study. Infect Dis Poverty. 2021;10(1):110. 34412700 10.1186/s40249-021-00893-6 PMC8374119

[7] Kim DS, Acharya D, Lee K et al. Awareness and work-related factors associated with scrub typhus: a case-control study from South Korea. Int J Environ Res Public Health. 2018;15(6):6. 10.3390/ijerph15061143 PMC6025028 29865144

[8] Wangrangsimakul T, Elliott I, Nedsuwan S et al. The estimated burden of scrub typhus in Thailand from national surveillance data (2003-2018). PLoS Negl Trop Dis. 2020;14(4):e0008233. 32287307 10.1371/journal.pntd.0008233 PMC7182275

[9] Wangrangsimakul T, Greer RC, Chanta C et al. Clinical characteristics and outcome of children hospitalized with scrub typhus in an area of endemicity. J Pediatric Infect Dis Soc. 2020;9(2):202–9. 30864670 10.1093/jpids/piz014 PMC7192406

[10] Chiangrai Provincial Statistical Report. In: Office CPSs (ed). Chiangrai, Thailand: National Statistical Office, 2021.

[11] Moonpanane K, Pitchalard K, Thepsaw J et al. Healthcare service utilization of hill tribe children in underserved communities in thailand: barriers to access. BMC Health Serv Res. 2022;22(1):1114. 36050759 10.1186/s12913-022-08494-1 PMC9438234

[12] Greer RC, Kanthawang N, Roest J et al. Vulnerability and agency in research participants’ daily lives and the research encounter: a qualitative case study of participants taking part in scrub typhus research in northern Thailand. PLoS One. 2023;18(1):e0280056. 36696400 10.1371/journal.pone.0280056 PMC9876277

[13] Cyril S, Smith BJ, Possamai-Inesedy A et al. Exploring the role of community engagement in improving the health of disadvantaged populations: a systematic review. Glob Health Action. 2015;8(1):29842. 26689460 10.3402/gha.v8.29842 PMC4685976

[14] O'Mara-Eves A, Brunton G, Oliver S et al. The effectiveness of community engagement in public health interventions for disadvantaged groups: a meta-analysis. BMC Public Health. 2015;15(1):129. 25885588 10.1186/s12889-015-1352-y PMC4374501

[15] Balcázar HG, de Heer H, Rosenthal L et al. A promotores de salud intervention to reduce cardiovascular disease risk in a high-risk Hispanic border population, 2005-2008. Prev Chronic Dis. 2010;7(2):A28. 20158973 PMC2831782

[16] Questa K, Das M, King R et al. Community engagement interventions for communicable disease control in low- and lower- middle-income countries: evidence from a review of systematic reviews. Int J Equity Health. 2020;19(1):51. 32252778 10.1186/s12939-020-01169-5 PMC7137248

[17] Skevington SM, Sovetkina EC, Gillison FB. A systematic review to quantitatively evaluate ‘Stepping Stones’: a participatory community-based HIV/AIDS prevention intervention. AIDS Behav. 2013;17(3):1025–39. 23128978 10.1007/s10461-012-0327-6

[18] Chiang Rai Highland People Development Center . Basic information on highland communities; 2016, unpublished.

[19] Wangrangsimakul T, Kanthawang N, Jaiboon P. Scrub typhus in northern Thailand. Zenodo, 2020. Available at 10.5281/zenodo.4084196 [accessed 25 April 2024].

[20] Wangrangsimakul T, Kanthawang N, Jaiboon P. Scrub Typhus Public Engagement in Nort hern Thailand—teaching flipchart in Thai and translation into English. 2023. zenodo.org, 10.5281/zenodo.8215181 [accessed 25 April 2024].

[21] Hartzler AL, Tuzzio L, Hsu C et al. Roles and functions of community health workers in primary care. Ann Fam Med. 2018;16(3):240–5. 29760028 10.1370/afm.2208 PMC5951253

[22] Wallerstein N, Oetzel JG, Duran B et al. Culture-centeredness in community-based participatory research: contributions to health education intervention research. Health Educ Res. 2019;34(4):372–88. 31237937 10.1093/her/cyz021 PMC6646947

[23] LaVigna GW, Christian L, Willis TJ. Developing behavioural services to meet defined standards within a national system of specialist education services. Pediatr Rehabil. 2005;8(2):144–55. 16089255 10.1080/13638490400024036

[24] Page TJ, Iwata BA, Reid DH. Pyramidal training: a large-scale application with institutional staff. J Appl Behav Anal. 1982;15(3):335–51. 7142060 10.1901/jaba.1982.15-335 PMC1308280

[25] Haens , sgen MJ, Xayavong T, Charoenboon N et al. The consequences of AMR education and awareness raising: outputs, outcomes, and behavioural impacts of an antibiotic-related educational activity in Lao PDR. Antibiotics. 2018;7(4):95. 10.3390/antibiotics7040095 PMC6316454 30388824

[26] Ma C-J, Oh G-J, Kang G-U et al. Differences in agricultural activities related to incidence of scrub typhus between Korea and Japan. Epidemiol Health. 2017;39(0):e2017051–0. 29121711 10.4178/epih.e2017051 PMC5790984

[27] Audet CM, Hamilton E, Hughart L et al. Engagement of traditional healers and birth attendants as a controversial proposal to extend the HIV health workforce. Current HIV/AIDS Reports. 2015;12(2):238–45. 25855337 10.1007/s11904-015-0258-8 PMC4430841

[28] Poudel KC, Jimba M, Joshi AB et al. Retention and effectiveness of HIV/AIDS training of traditional healers in far western Nepal. Trop Med Int Health. 2005;10(7):640–6. 15960702 10.1111/j.1365-3156.2005.01443.x

