# Supplementary material for: Community engagement around scrub typhus in northern Thailand: a pilot project
Source: Trans R Soc Trop Med Hyg. 2024 May 6;118(10):666–73. doi: 10.1093/trstmh/trae028 (PMC11443339; doi:10.1093/trstmh/trae028)
Supplement: trae028_Supplemental_File [file trae028_supplemental_file.docx]

# Community Engagement around Scrub Typhus in Northern Thailand

# Supplementary materials and Appendices

Supplementary Table S1 - Scrub typhus knowledge questionnaire (correct answer in bold).

| Question | Answer | Frequency n/N (%) |
| --- | --- | --- |
| What is the name of scrub typhus in Thai? | 1). Ma-La-Ria  **2). Kai-Rak-Sat-Yai**  3). Chee-Noo  4). Kai-Luat-Aok  5). I don’t know/ not sure | 1/133 (0.8)  91/133 (68.4)  12/133 (9.0)  6/133 (4.5)  23/133 (17.3) |
| What pathogen causes scrub typhus? | 1). Fungus  2). Parasite  **3). Bacteria**  4). Virus  5). I don’t know/ not sure | 12/133 (9.0)  18/133 (13.5)  39/133 (29.3)  30 (22.6)  34 (25.6) |
| What is the vector of scrub typhus? | 1). Dog  **2). Mite**  3). Mosquito  4). Cockroach  5). I don’t know/ not sure | 5/133 (3.8)  65/133 (48.9)  22/133 (16.5)  9/133 (6.8)  32/133 (24.1) |
| Which animal does the vector usually feed on? | **1). Rats**  2). Pigs  3). Human  4). Dogs  5). I don’t know/ not sure | 60/130 (46.2)  4/130 (3.1)  16/130 (12.3)  15/130 (11.5)  35/130 (26.9) |
| What habitat is NOT associated with scrub typhus? | **1). Schools**  2). Forest  3). Coffee/tea plantations  4). Rice field  5). I don’t know/ not sure | 65/133 (48.9)  19/133 (14.3)  7/133 (5.3)  11/133 (8.3)  31/133 (23.3) |
| Which patient is likely to have scrub typhus? | 1). Mr. Khampon has had chest pain and dyspnoea for 3 days.  2). Mrs. Amphorn has had dysuria and lower abdominal pain for 1 day.  **3). Mr.Suphap has fever, headache and eschar for 5 days.**  4). Porjai a young boy has had a rash on his back since yesterday.  5). I don’t know/ not sure | 10/126 (7.9)  2/126 (1.6)  78/126 (61.9)  8/126 (6.4)  28/126 (22.2) |
| Which picture shows the skin lesion (eschar) associated with scrub typhus infection? | 1). Ringworm  **2). Eschar**  3). Psoriasis  4). Herpes vesicles  5). I don’t know/not sure | 16/129 (12.4)  66/129 (51.2)  6/129 (4.7)  10/129 (7.8)  31/129 (24.0) |
| *For HCWs*  Which medicine is used to treat scrub typhus?  *For CHVs*  Which medicine is used to treat scrub typhus? | 1). Amoxicillin  2). Co-amoxiclav  **3). Doxycycline**  4). Dicloxacillin  5). I don’t know/ not sure  **1). Antibiotics**  2). Paracetamol  3). Anti-diarrhoeals  4). Anti-emetics  5). I don’t know/not sure | 1/17 (5.9)  1/17 (5.9)  6/17 (35.3)  3/17 (17.7)  6/17 (35.3)  59/115 (51.3)  24/115 (20.9)  0  3/115 (2.6)  29/115 (25.2) |
| *For HCWs*  During 2008-2017, which sub-district in Mueang Chiang Rai district reported the most scrub typhus patients?  *For CHVs*  Which province reported the most scrub typhus patients in Thailand? | 1). Mae Korn  2). Tha Sai  3). Doi Lan  **4). Huay Chompoo**  5). I don’t know/ not sure  1). Payao  2). Chiang Mai  **3). Chiang Rai**  4). Nan  5). I don’t know/not sure | 1/17 (5.9)  0  0  9/17 (52.9)  7/17 (41.2)  1/116 (0.9)  17/116 (14.7)  38/116 (32.8)  17/116 (14.7)  43/116 (37.1) |
| Which of the following occupation has the highest risk for scrub typhus? | 1). Police  2). Teacher  3). Doctor  **4). Farmer**  5). I don’t know/ not sure | 0  0  3/134 (2.2)  108/134 (80.6)  23/134 (17.2) |
| How can you prevent catching scrub typhus? | 1). Lie down to rest on the ground at break time  2). Vaccination  **3). Wear protective clothing**  4). Exercising and eating healthy foods.  5). I don’t know/ not sure | 8/134 (6.0)  32/134 (23.9)  51/134 (38.1)  17/134 (12.7)  26/134 (19.4) |
| Which of the following is NOT recommended? | 1). Take a shower soon after returning home from agricultural work.  2). Wear fully-covered clothing, long-sleeved shirts and long pants, even on hot days.  **3). Buy medicine at the pharmacy when you feel unwell and think you have scrub typhus.**  4) Clean and maintain waste areas and areas around the house regularly.  5). I don’t know/ not sure | 10/131 (7.6)  11/131 (8.4)  70/131 (53.4)  17/131 (13.0)  23 (17.6) |
| Which of the following is correct regarding management of scrub typhus? | **1). If you have any symptoms that suggest scrub typhus e.g. fever and eschar after working in the field, plantation or forest, seek medical help.**  2). Ignore symptoms of scrub typhus as it rarely needs treatment.  3). Take medication every day to prevent scrub typhus.  4). I can use herbal or traditional medicine to treat scrub typhus.  5). I don’t know/ not sure | 102/133 (76.7)  3/133 (2.3)  5/133 (3.8)  2/133 (1.5)  21/133 (15.8) |

Supplementary Table S2 Multivariable logistic regression on paired difference between pre- and post-session scrub typhus knowledge scores

| Variable | Coefficient (95% CI) | P value |
| --- | --- | --- |
| Pre-session knowledge score | -0.93 (-0.99 to -0.87) | <0.001 |
| Education  Less than primary school  Primary school certificate  High school certificate  Higher education | Reference  -0.38 (-1.08 to 0.33)  -0.23 (-0.90 to 0.44)  0.001 (-0.99 to 1.00) | 0.290  0.490  0.988 |
| Ethnicity  Thai  Akha  Lahu  Karen  Other | Reference  -0.15 (-0.73 to 0.42)  -0.14 (-0.76 to 0.48)  0.02 (-0.69 to 0.72)  -0.20 (-1.01 to 0.61) | 0.599  0.663  0.964  0.627 |
| Belief that scrub typhus is a problem in this area  Agree  Disagree  Don’t know or neutral | Reference  0.06 (-0.32 to 0.44)  -0.00 (-0.52 to 0.52) | 0.751  0.993 |
| Healthcare worker (rather than CHV) | 0.14 (-0.64 to 0.92) | 0.723 |
| PCU for training  PCU 1  PCU 2  PCU 3  PCU 4  PCU 5 | Reference  -0.70 (-1.35 to -0.04)  -0.25 (-0.90 to 0.40)  0.07 (-0.49 to 0.63)  0.15 (-0.53 to 0.83) | 0.038  0.439  0.806  0.662 |
| PCU: Primary care unit; CHV: Community health volunteer | | |

Supplementary Table S3 - Univariate analysis of paired difference between pre- and post-session scrub typhus knowledge scores

|  | Number of participants, n/N (%) | Score difference  (p25, p75) | p-value^a^ |
| --- | --- | --- | --- |
| n | 134 |  |  |
| Healthcare worker role |  |  | <0.001 |
| Community health volunteer | 117/134 (87.3%) | 5 (4, 8) |  |
| Healthcare worker | 17/134 (12.7%) | 2 (1, 4) |  |
| Age group, n (%) |  |  |  |
| <35 | 35/134 (26.1%) | 5 (2, 8) |  |
| 35 to 39 | 23/134 (17.2%) | 6 (4, 8) |  |
| 40 to 49 | 44/134 (32.8%) | 4 (3, 6) |  |
| >=50 | 32/134 (23.9%) | 5 (3.5, 9) |  |
| Educational level |  |  | <0.001 |
| Less than primary school | 9/133 (6.8%) | 9 (8, 10) |  |
| Primary school | 33/133 (24.8%) | 5 (4, 8) |  |
| High school | 69/133 (51.9%) | 5.0 (3, 8) |  |
| Higher education | 22/133 (16.5%) | 2.5 (1, 4) |  |
| Ethnicity, n (%) |  |  | <0.001 |
| Thai | 41/134 (30.6%) | 4 (2, 6) |  |
| Akha | 37/134 (27.6%) | 7 (5, 10) |  |
| Lahu | 32/134 (23.9%) | 5 (3.5, 8) |  |
| Karen | 16/134 (11.9%) | 4 (2.5, 4.5) |  |
| Other | 8/134 (6.0%) | 3.5 (2, 9) |  |
| PCU |  |  | 0.006 |
| PCU 1 | 22/134 (16.4%) | 6 (5, 8) |  |
| PCU 2 | 37/134 (27.6%) | 4 (3, 7) |  |
| PCU 3 | 28/134 (20.9%) | 4 (2, 5.5) |  |
| PCU 4 | 27/134 (20.1%) | 8 (4, 10) |  |
| PCU 5 | 20/134 (14.9%) | 4 (3, 7.5) |  |
| Belief that scrub typhus is a problem in the area |  |  | <0.001 |
| Yes | 52/131 (39.7%) | 3.5 (2, 5) |  |
| No | 62/131 (47.3%) | 6 (4, 10) |  |
| Neutral/Don't know | 17/131 (13.0%) | 5 (4, 7) |  |
| Self-reported awareness of scrub typhus pre-session |  |  | <0.001 |
| No | 66/133 (49.6%) | 6.5 (4, 9) |  |
| Yes | 67/133 (50.4%) | 4 (2, 6) |  |
| Self-reported experience of scrub typhus patients pre-session |  |  | 0.007 |
| No | 109/132 (82.6%) | 5 (4, 8) |  |
| Yes | 23/132 (17.4%) | 2 (1, 7) |  |
| Thai language ability |  |  | 0.11 |
| Able to speak & read Thai | 102/107 (95.3%) | 5 (3, 8) |  |
| Unable to speak & read Thai | 5/107 (4.7%) | 8 (6, 10) |  |
| Workplace, n (%) |  |  | 0.007 |
| PCU 1 | 25/134 (18.7%) | 6 (5, 7) |  |
| PCU 2 | 30/134 (22.4%) | 5 (4, 7) |  |
| PCU 3 | 28/134 (20.9%) | 4 (2, 5.5) |  |
| PCU 4 | 27/134 (20.1%) | 8 (4, 10) |  |
| PCU 5 | 24/134 (17.9%) | 4 (2, 5) |  |
| p25, p75: 25^th^ and 75^th^ percentile  PCU: Primary care unit  a *p-value calculated using Wilcoxon rank-sum (2 groups) or Kruskal-Wallis (>2 groups) test* | | | |
